# Exploring Health Literacy, Anxiety, and Perception Among Nursing and Allied Health Students in Turkey: A Cross‐Sectional Study

**DOI:** 10.1111/jep.70491

**Published:** 2026-06-09

**Authors:** Akgün Yeşiltepe, Ferhat Daşbilek, Mehmet Osman Ayhan

**Affiliations:** ^1^ Munzur University Faculty of Health Science Tunceli Turkey

**Keywords:** health anxiety, health literacy, health perception, health sciences students

## Abstract

**Background:**

Health literacy, anxiety, and perception directly affect healthcare students' readiness for professional roles. Assessing these factors is crucial to enhance both their well‐being and patient care quality. This study examined health literacy, health anxiety, and health perception among Health Sciences students and their links to socio‐demographic factors.

**Methods:**

A cross‐sectional and descriptive design was used in accordance with the STROBE guidelines. The sample, consisting of 258 students from Munzur University Faculty of Health Sciences, was selected using stratified and simple random sampling methods. Data were collected using a Descriptive Information Form, the Health Literacy Scale, the Health Anxiety Inventory, and the Health Perception Scale. Descriptive statistics, Mann−Whitney *U*, Kruskal−Wallis tests with Bonferroni correction, and Spearman's correlation analysis were conducted using SPSS 26 and JASP 0.19.

**Results:**

Students demonstrated inadequate health literacy (17.75 ± 11.37) and moderate health perception (43.43 ± 6.25); the mean health anxiety score was 18.78 ± 6.14. A weak but statistically significant positive correlation was found between health literacy and health anxiety (*r* = 0.225, *p* < 0.05) and between health perception and health anxiety (*r* = 0.215, *p* < 0.05); however, no significant correlation was observed between health literacy and health perception (*r* = 0.090, *p* > 0.05). Health perception revealed statistically significant differences according to maternal education level, place of residence, and frequency of reading health‐related research. Health literacy significantly differed by academic department, year of study, perceived health status, and perceived usefulness of online health information. Health anxiety varied significantly by the presence of chronic illness, chronic illness status, perceived health status, medication use, and alcohol/tobacco consumption.

**Conclusion:**

The findings highlight the complex interplay between cognitive (health literacy), emotional (health anxiety), and perceptual (health perception) dimensions of student health. Promoting digital health literacy and providing counselling services may help reduce health anxiety and enhance self‐awareness among health sciences students.

## 1 Introductıon

The protection and promotion of health is a dynamic process aimed at enhancing individuals' control over their own health, enabling them to make informed health‐related decisions, and improving their quality of life [1]. A fundamental component of this process is the adoption of healthy lifestyle behaviours. Structuring individuals' lifestyles in accordance with health principles contributes not only to the protection of personal health but also to the improvement of public health [2]. Healthy lifestyle behaviours encompass taking responsibility for maintaining and promoting one's own health, coping effectively with stress, monitoring one's health status, engaging in regular physical activity, and adopting balanced dietary habits [3]. In today's information age, increased access to health‐related information and the growing need to critically evaluate such information have brought the concept of health literacy to the forefront [4, 5]. Health literacy refers to an individual's ability to access, comprehend, evaluate, and effectively use basic health information and services. It is also a broad competency area that encompasses the intergenerational transmission of health knowledge [6, 7]. Students of health sciences, as future healthcare providers, constitute a critical group that is expected to possess a high level of health literacy to interpret and apply accurate and up‐to‐date health information [8]. In today's digitalised information environment, the process of accessing health information is intertwined with health literacy, fact‐checking abilities, and critical thinking skills. In this context, low levels of health literacy among students may lead to distrust in the healthcare system and consequently increase health anxiety [9]. Health anxiety refers to a psychological state characterised by excessive concern or worry related to one's health status and is often accompanied by heightened sensitivity to bodily sensations and perceived health threats [10, 11]. Although a certain level of concern about health may encourage preventive health behaviours and medical consultation when necessary, excessive anxiety may negatively affect psychological well‐being and daily functioning. Individuals experiencing high levels of health anxiety may display different behavioural patterns, such as postponing healthcare visits due to fear of receiving unfavourable diagnoses or repeatedly seeking reassurance through frequent medical consultations and health monitoring behaviours. Students enroled in health sciences programmes may experience health anxiety differently compared to other student groups due to increased exposure to medical terminology, disease symptoms, and clinical scenarios throughout their education [9]. Continuous exposure to disease‐related information may increase awareness of potential health risks, which may influence both perceived vulnerability and anxiety responses.

Health‐related behaviours are not shaped solely by cognitive competencies such as health literacy but are also influenced by emotional and psychological processes. Factors such as emotion regulation abilities, psychological distress, coping strategies, and behavioural tendencies may affect how individuals interpret health‐related stimuli and how they respond to perceived health risks. Psychological characteristics have been shown to influence individuals' health awareness, decision‐making processes, and behavioural outcomes across different populations. Research conducted among university students has demonstrated that emotional eating behaviours are significantly associated with eating behaviour disorders and difficulties in emotion regulation, suggesting that emotional processes may shape responses to health‐related information and health behaviours [12]. Similarly, studies examining attitudes and knowledge regarding functional foods have indicated that behavioural preferences related to health are influenced not only by cognitive awareness but also by psychological and behavioural determinants [13]. Furthermore, research conducted among individuals diagnosed with type 2 diabetes has shown that psychological factors such as depression and alexithymia significantly influence emotional eating behaviours, highlighting the role of emotional processes in shaping health‐related perceptions and behaviours [14]. These findings indicate that psychological processes may interact with health literacy in influencing individuals' health perceptions and levels of health anxiety.

Health perception represents another important concept associated with health‐related decision‐making processes. It refers to individuals' subjective evaluation of their own health status, which develops through the interaction of personal experiences, beliefs, expectations, and emotional interpretations [15]. Health perception does not solely reflect objective medical conditions but also includes personal interpretations influenced by social environment, psychological characteristics, and individual health experiences [16]. Individuals who perceive their health positively are more likely to adopt preventive health behaviours, demonstrate higher levels of motivation to maintain well‐being, and show greater psychological resilience when facing health‐related challenges. Previous research has demonstrated that higher levels of health literacy are associated with more positive health perceptions because individuals with adequate health knowledge are better able to interpret symptoms accurately and evaluate health risks realistically [14]. Strengthening health perception has also been linked to improved professional competence among health sciences students as well as increased psychological resilience [17]. In addition, studies suggest that individuals with more positive perceptions of their health may experience lower levels of health anxiety because they feel more capable of understanding and managing potential health threats [18, 19].

The university period is characterised by substantial developmental changes involving academic responsibilities, social adaptation, financial challenges, and increased independence in decision‐making processes [20]. During this transitional stage, individuals begin to take greater responsibility for maintaining their own health and developing long‐term behavioural patterns. Health behaviours acquired during university years often persist into adulthood and influence future health outcomes. For students studying in health‐related disciplines, developing positive health‐related attitudes is particularly important because these individuals are expected to serve as role models in promoting health awareness and supporting preventive health practices within society [2, 5]. Health sciences students are required not only to develop theoretical knowledge but also to acquire skills that enable them to guide individuals and communities in making informed health decisions. However, the predominantly disease‐focused structure of many health sciences curricula may lead students to focus primarily on pathological conditions rather than preventive health approaches. Despite the increasing importance of promoting health awareness among future healthcare professionals, limited research has examined how health‐oriented and disease‐oriented educational approaches influence students' health literacy, health perception, and health anxiety levels.

Therefore, the present study aims to determine the levels of health literacy, health anxiety, and health perception among students enroled in the Faculty of Health Sciences and to examine the relationships among these variables. Evaluating both cognitive competencies and psychological factors related to health may contribute to a more comprehensive understanding of the factors influencing health awareness and health‐related decision‐making processes among future healthcare professionals. The findings of this study are expected to provide evidence that may support the development of educational strategies aimed at improving both professional competence and personal well‐being among health sciences students.

### 1.1 Research Questions

- What are the levels of health literacy, health anxiety, and health perception among students?
- Is there a relationship between students' health literacy, health anxiety, and health perception?
- Do health literacy, health anxiety, and health perception levels differ according to socio‐demographic variables?

## 2 Methods

### 2.1 Study Population and Sample

This study was conducted using a cross‐sectional and descriptive design in order to examine the relationships among health literacy, health anxiety, and health perception among students enroled in health‐related academic programmes. Cross‐sectional questionnaire‐based designs are frequently used in studies investigating psychological determinants of health‐related behaviours because they enable the simultaneous evaluation of cognitive, emotional, and behavioural variables within a defined population. Such designs are considered appropriate for identifying potential associations among variables and for generating evidence regarding factors influencing health awareness and behavioural tendencies.

The population of the study consisted of students enroled in departments affiliated with the Faculty of Health Sciences at Munzur University during the 2022–2023 academic year (*N* = 777). The required sample size was calculated using the known population formula, considering the population size, confidence level, and acceptable margin of error.

$$n=\frac{N\cdot Z^{2}\cdot p\cdot (1-p)}{E^{2}\cdot (N-1)+Z^{2}\cdot p\cdot (1-p)}$$

A stratified sampling approach was applied in order to ensure adequate representation of each academic department within the Faculty of Health Sciences. Each department was treated as a separate stratum, and proportional allocation was applied based on the number of students enroled in each department relative to the total population. Within each stratum, simple random sampling was performed using official student lists as the sampling frame. Student lists were obtained from departmental administrations, and eligible participants were randomly selected using the Randomiser software [21]. This procedure ensured that each student had an equal probability of being selected and reduced the risk of sampling bias.

The survey link was sent directly and exclusively to randomly selected students. The questionnaire link was not distributed to the entire student population in order to maintain adherence to probability sampling principles and to reduce self‐selection bias. The final sample consisted of 258 participants, including 79 students from the Nursing department, 43 students from Midwifery, 53 students from Child Development, 36 students from Nutrition and Dietetics, 25 students from Emergency and Disaster Management, and 22 students from Social Work.

Participant flow was documented to enhance transparency of the sampling process. A total of 258 students were invited to participate in the study, and all invited students completed the questionnaire. No questionnaires were excluded due to incomplete or incorrectly filled responses. Therefore, the final dataset consisted of responses obtained from 258 participants.

Post‐hoc power analysis was conducted to evaluate whether the sample size was sufficient to detect statistically meaningful relationships among variables. The analysis indicated approximately 95.9% statistical power (*α* = 0.05) to detect a correlation coefficient of *r* = 0.225, suggesting that the sample size was adequate for identifying significant relationships among study variables.

The study was reported in accordance with the Strengthening the Reporting of Observational Studies in Epidemiology (STROBE) guidelines in order to ensure methodological transparency and completeness.

### 2.2 Inclusion Criteria

Participants were required to be aged 18 years or older and actively enroled in one of the departments affiliated with the Faculty of Health Sciences during the data collection period. Students were expected to have sufficient cognitive ability to understand the study information and provide informed consent voluntarily. Only individuals who agreed to participate after reviewing the study information were included.

### 2.3 Exclusion Criteria

Students who declined to participate, withdrew from the study before completing the questionnaire, or submitted forms containing incomplete or inconsistent responses were excluded from the study.

### 2.4 Data Collection Tools

Data were collected using a structured questionnaire consisting of the Descriptive Information Form, the Health Perception Scale, the Health Anxiety Inventory, and the Turkey Health Literacy Scale‐32 (TSOY‐32). These instruments were selected because they have demonstrated validity and reliability in previous studies and are frequently used to assess psychological and cognitive determinants of health‐related behaviours.

### 2.5 Descriptive Information Form

The Descriptive Information Form was developed by the researchers following a review of relevant literature [4, 5, 8, 12, 15, 17]. The form consists of 21 items designed to obtain information regarding socio‐demographic characteristics, including age, gender, marital status, educational background, and other variables that may influence health‐related perceptions and behaviours.

### 2.6 Health Perception Scale

The Health Perception Scale was used to evaluate individuals' beliefs, attitudes, and perceptions regarding their own health. The scale was originally developed by Diamond [22], and its Turkish validity and reliability were established by Kadıoğlu and Yıldız [23]. The instrument consists of 15 items and includes four subdimensions: locus of control, self‐awareness, certainty, and importance of health.

Items 2, 3, 4, 6, 7, 8, 12, 13, and 15 are negatively worded and were reverse scored during analysis. Responses are recorded on a five‐point Likert scale ranging from 1 (strongly disagree) to 5 (strongly agree). Total scores range from 15 to 75, with higher scores indicating a higher level of perceived health. In the present study, internal consistency reliability was found to be acceptable (Cronbach's *α* = 0.73).

### 2.7 Health Anxiety Inventory

The Health Anxiety Inventory was used to assess the level of anxiety related to individuals' health status. The scale was originally developed by [24], and its Turkish validity and reliability were established by [25]. The inventory consists of 18 items scored on a four‐point Likert scale ranging from 0 to 3. Higher total scores indicate higher levels of health anxiety.

The inventory includes two subdimensions. The first subdimension consists of 14 items assessing bodily sensitivity and concerns related to physical symptoms (somatic dimension). The second subdimension includes four items evaluating cognitive concerns regarding possible negative health outcomes (negative consequences dimension).

The original scale demonstrated high reliability, with a Cronbach's alpha value of 0.91. In the present study, internal consistency reliability was found to be acceptable (Cronbach's *α *= 0.77).

### 2.8 TSOY‐32

Participants' health literacy levels were evaluated using the TSOY‐32. This instrument was developed by the Ministry of Health of the Republic of Turkey in collaboration with the Department of Public Health at Adnan Menderes University Faculty of Medicine [26].

The scale consists of 32 items designed to measure individuals' ability to access, understand, evaluate, and apply health‐related information. Responses are recorded on a five‐point Likert scale ranging from ‘very easy’ to ‘very difficult’ and ‘don't know’.

Based on the European Health Literacy Survey (HLS‐EU), responses were transformed into a standardised index ranging from 0 to 50, and health literacy levels were categorised as inadequate (0–25), problematic or limited (25–33), sufficient (33–42), and excellent (42–50). This classification provides a structured framework for evaluating individuals' ability to effectively use health‐related information. In the present study, the scale demonstrated high internal consistency (Cronbach's *α* = 0.96).

### 2.9 Data Collection

Data was collected via an online survey link, distributed only through WhatsApp and in‐class communication, and sent to students selected using stratified random sampling. It was not shared as an open invitation to all students. Prior to participation, students were informed about the purpose and scope of the study and provided informed consent by selecting the statement ‘I voluntarily agree to participate in this study’.

To ensure confidentiality and prevent duplicate responses, the online survey system was configured to allow only one submission per participant. Only the researcher had access to the dataset through a secure Google account. The average time required to complete the questionnaire ranged between approximately 5 and 10 min.

### 2.10 Data Analysis

Statistical analyses were performed using SPSS version 26, JASP version 0.19, and G*Power version 3.1 software. Initially, descriptive statistics were calculated in order to summarise the characteristics of the dataset. Frequency, percentage, minimum and maximum values, median, mean, and standard deviation values were computed for relevant variables. Prior to selecting appropriate inferential statistical tests, the distributional characteristics of the variables were examined. Normality was evaluated using both statistical and graphical approaches. The normality of the data distribution was assessed based on skewness and kurtosis values. Skewness and kurtosis values falling outside the commonly accepted range of −1.5 to +1.5 were interpreted as indicators of deviation from normal distribution. The combined results of statistical tests and graphical evaluations indicated that the variables did not meet the assumptions required for parametric analysis. Therefore, non‐parametric statistical methods were used.

The Mann–Whitney *U* test was used to compare differences between two independent groups. The Kruskal–Wallis test was used to compare differences among more than two independent groups. When statistically significant differences were identified using the Kruskal–Wallis test, Bonferroni‐adjusted post‐hoc multiple comparison tests were conducted in order to determine which specific groups differed significantly from one another. Spearman's rank‐order correlation analysis was used to examine the relationships among health literacy, health perception, and health anxiety variables. Spearman correlation analysis is recommended when data do not demonstrate normal distribution and when relationships between variables are assessed using ranked values rather than raw scores. This method allows the identification of monotonic relationships without requiring assumptions of normal distribution or linearity. The use of cross‐sectional survey methodology and non‐parametric statistical analysis is consistent with previous studies examining psychological determinants of health‐related behaviours among university students [13]. A confidence interval of 95% was adopted for all statistical analyses, and statistical significance was accepted as *p* < 0.05.

## 3 Results

Among the students who participated in the study, 30.6% were enroled in the Nursing Department, and 33.7% were first‐year students. Regarding parental education levels, the majority of both mothers (%38.8) and fathers (58.1) were primary school graduates. More than half of the participants (53.5%) reported that their income exceeded their expenses, and 89.1% indicated that they did not have a chronic illness. In terms of perceived health status, 53.9% rated their health as moderate. While 77.5% reported not using any medications, 94.6% stated that they regularly read health‐related research. Additionally, 77.1% found health‐related information obtained from the internet to be useful. The proportion of students who reported using alcohol and tobacco was 15.9% (Table 1) (Figure 1).

**Table 1:** Descriptive characteristics of the students participating in the study (*n* = 258).

Variables		*n*	%
Department	Nursing	79	30.6
	Midwifery	43	16.7
	Child development	53	20.5
	Emergency and disaster management	25	9.7
	Social work	22	8.5
	Nutrition and dietetics	36	14.0
Academic year	1st year	87	33.7
	2nd year	61	23.6
	3rd year	68	26.4
	4th year	42	16.3
Mother's education level	Illiterate	99	38.4
	Literate	34	13.2
	Primary school	100	38.8
	High school and above	25	9.7
Father's education level	Illiterate	30	11.6
	Literate	12	4.7
	Primary school	150	58.1
	High school and above	66	25.6
Income status	Income > Expenses	138	53.5
	Income = Expenses	102	39.5
	Income < Expenses	18	7.0
Current residence	With family	113	43.8
	Dormitory	139	53.9
	Other	6.0	2.3
Chronic illness	Yes	28	10.9
	No	230	89.1
Perceived health status	Good	104	40.3
	Moderate	139	53.9
	Poor	15	5.8
Medication use	Yes	58	22.8
	No	200	77.5
Reads health‐related research	Yes	244	94.6
	No	14	5.4
Finds online health information useful	Yes	199	77.1
	No	59	22.9
Alcohol or tobacco use	Yes	41	15.9
	No	217	84.1
Total		258	100

**FİGURE 1:**
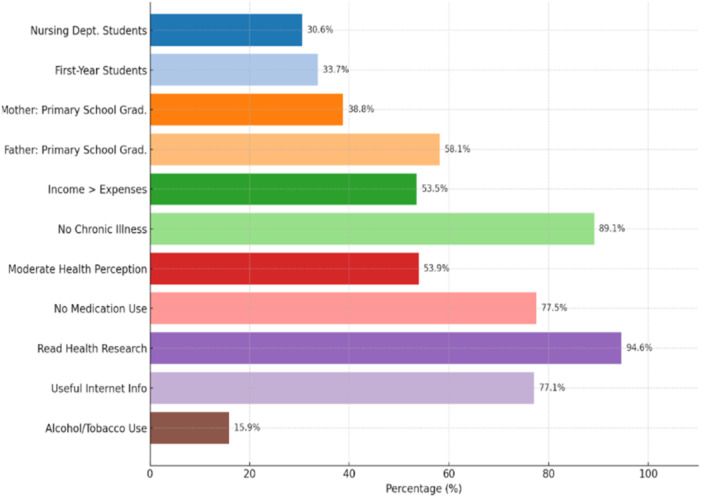
Descriptive characteristics of the participating students.

Descriptive statistics showed that the mean TSOY‐32 health literacy index score was 17.75 ± 11.37, which falls within the inadequate health literacy category (0–25). The mean Health Anxiety score was 18.78 ± 6.14 (possible range: 0–54). The mean Health Perception score was 43.43 ± 6.25 (possible range: 15–75), suggesting a moderate level of perceived health. The findings of the Spearman correlation analysis revealed a statistically significant yet weak positive relationship between health literacy and health anxiety (*r* = 0.225, *p* < 0.05). This result suggests that individuals with higher levels of health literacy may experience slightly greater health anxiety. Likewise, health perception was found to be weakly and positively correlated with health anxiety (*r* = 0.215, *p* < 0.05), indicating that an increase in health perception may be accompanied by a slight increase in health anxiety levels. On the other hand, the correlation between health literacy and health perception was not statistically significant (*r* = 0.090, *p* > 0.05), suggesting the absence of a meaningful linear association between these variables. Taken together, these results indicate that while health literacy and health perception are each modestly associated with health anxiety, they do not appear to be significantly related to one another. Based on these findings, students demonstrated low levels of health literacy and health anxiety, while their perceived health status was at a moderate level (Table 2). The scatter and density plots illustrating the relationships between the variables are presented below (Figure 2).

**Table 2:** Correlation analysis, means, and reliability results of the health literacy, health anxiety, and health perception scales.

Scales	1	2	3	Mean ± SD	Cronbach alpha (*α*)
Health literacy (1)	1	0.225*	0.090	17.75 ± 11.37	0.96
Health anxiety (2)	—	1	0.215*	18.78 ± 6.14	0.77
Health perception (3)	—	—	1	43.43 ± 6.25	0.73

*Note:* Values are Spearman's rho. *p* < 0.05.

**FİGURE 2:**
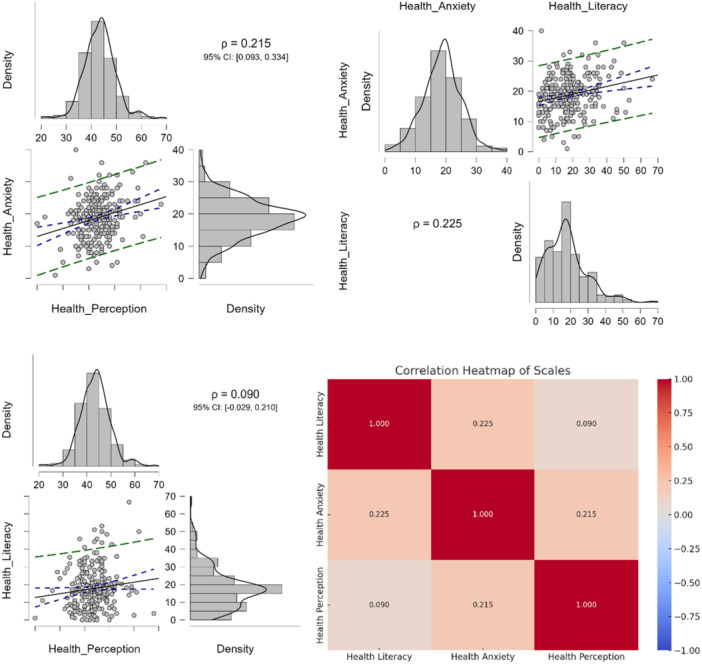
Correlation, scatter and density plot showing the relationships among the scales.

Analysis of participants' health perception scores by socio‐demographic variables revealed statistically significant differences based on maternal education level, current place of residence, and frequency of reading health‐related research (*p* < 0.05). Students who regularly read health‐related research had significantly higher health perception scores (43.71 ± 6.19) compared to those who did not (38.64 ± 5.55). Post‐hoc Bonferroni analysis indicated that students whose mothers were literate but had no formal education had significantly higher health perception scores than those whose mothers were either illiterate or only primary school graduates (Table 3).

**Table 3:** Comparison of scale mean scores according to the descriptive characteristics of the students (*n* = 258).

		Health perception scale			Health anxiety scale			Health literacy scale		
Variables		XX ± SD	Median	U/KW/p/bonferroni	XX ± SD	Median	U/KW/p/bonferroni	XX ± SD	Median	U/KW/p/bonferroni
Department***	Nursing (a)	43.87 ± 5.69	4300	6.029 0.303	19.41 ± 4.90	19.00	7.945 0.159	19.36 ± 11.50	18.22	14.068 0.015* **e** > **f**
	Midwifery (b)	44.51 ± 5.88	44.00		19.93 ± 5.74	20.00		17.18 ± 12.03	15.62	
	Child Development (c)	42.24 ± 5.28	43.00		17.50 ± 5.93	18.00		16.36 ± 9.94	15.62	
	Emergency and Disaster Management (d)	44.72 ± 8.93	46.00		17.84 ± 8.19	17.00		16.12 ± 12.32	15.62	
	Social Work (e)	42.63 ± 6.70	41.50		19.63 ± 6.56	20.00		24.24 ± 11.71	21.09	
	Nutrition and Dietetics (f)	42.55 ± 6.63	44.00		18.02 ± 7.30	17.00		14.12 ± 9.84	13.54	
Academic Year***	1st Year (a)	43.65 ± 5.02	44.00	0.822 0.844	19.19 ± 6.22	19.00	4.592 0.204	19.22 ± 10.60	17.70	12.529 0.006* **a** > **d, b** > **d**
	2nd Year (b)	42.78 ± 5.29	43.00		17.55 ± 5.91	18.00		18.45 ± 10:53	17.70	
	3rd Year (c)	43.69 ± 8.10	44.00		19.66 ± 6.43	20.00		18.29 ± 13.33	16.14	
	4th Year (d)	43.52 ± 6.57	43.00		18.28 ± 5.70	19.00		12.81 ± 9.54	10.93	
Mother's Education Level***	Illiterate (a)	43.21 ± 5.65	43.00	12.442 0.006* **b** > **c, b** > **a**	18.59 ± 5.99	19.00	0.421 0.936	17.95 ± 10.26	16.66	0.206 0.977
	Literate(b)	46.50 ± 7.75	47.00		19.05 ± 5.43	19.00		18.97 ± 15.46	17.44	
	Primary School (c)	42.72 ± 6.28	43.00		18.54 ± 6.77	19.00		18.02 ± 11.63	17.44	
	High School and Above (d)	43.04 ± 5.19	43.00		20.12 ± 5.06	20.00		14.22 ± 7.25	14.06	
Father's Education Level***	Illiterate (a)	43.16 ± 6.41	44.00	0.941 0.815	19.40 ± 7.75	19.50	2.792 0.425	20.57 ± 9.98	20.57	5.672 0.129
	Literate (b)	41.50 ± 8.18	44.50		20.75 ± 5.11	20.00		22.30 ± 14.61	17.18	
	Primary School (c)	43.40 ± 6.05	43.00		18.52 ± 5.92	19.00		17.02 ± 11.04	16.14	
	High School and Above (d)	43.98 ± 6.32	44.0		18.74 ± 6.03	19.00		17.29 ± 11.94	15.62	
Income Status***	Income > Expenses (a)	43.56 ± 6.59	44.00	1.427 0.490	19.66 ± 6.32	19.00	9.624 0.008* **c** > **b**	18.64 ± 10.36	17.18	4.321 0.115
	Income = Expenses (b)	43.51 ± 5.99	43.00		17.14 ± 5.97	17.00		17.15 ± 12.17	14.84	
	Income < Expenses (c)	42.00 ± 5.06	41.50		19.77 ± 4.25	20.50		14.35 ± 11.95	15.62	
Current Residence***	With Family	42.62 ± 5.01	43.00	7.732 0.021*	18.53 ± 6.16	19.00	1.706 0.426	17.89 ± 10.41	16.66	2.222 0.329
	Dormitory	44.30 ± 19.09	44.00		19.09 ± 6.20	19.00		17.80 ± 12.14	16.66	
	Other	38.66 ± 6.56	36.00		16.33 ± 4.13	16.50		12.32 ± 10.68	10.15	
Chronic Illness**	Yes	44.53 ± 7.09	44.00	2917.00 0.416	23.10 ± 5.82	23.50	1783.00 0.000*	18.9111.08	17.70	−0.672 0.502
	No	43.30 ± 6.15	43.00		18.25 ± 5.98	11.42		17.61 ± 11.42	16.14	
Perceived Health Status***	Good (a)	43.07 ± 6.57	43.00	2.520 0.284	16.64 ± 5.74	17.00	24.091 0.000* **c** > **a, c** > **b**	15.55 ± 10.47	15.62	5.977 0.050*
	Moderate (b)	43.79 ± 5.87	44.00		19.84 ± 5.41	20.00		19.22 ± 11.54	17.70	
	Poor(c)	42.60 ± 7.53	42.00		23.73 ± 9.49	26.00		19.34 ± 13.92	16.14	
Medication Use**	Yes	43.82 ± 6.61	44.00	5564.50 0.786	21.08 ± 6.54	21.50	4122.00 0.001*	18.22 ± 12.01	16.40	−0.128 0.898
	No	43.32 ± 6.16	43.00		18.11 ± 5.87	18.50		17.61 ± 11.20	16.66	
Reads Health‐Related Research**	Yes	43.71 ± 6.19	44.00	941.00 0.005*	18.97 ± 6.10	19.00	1207.500 0.065	17.66 ± 11.30	16.66	−0.302 0.763
	No	38.64 ± 5.55	40.00		15.35 ± 6.03	17.00		19.38 ± 12.80	16.40	
Finds online health information useful**	Yes	43.71 ± 6.19	44.00	5305.00 0.261	18.70 ± 6.25	19.00	−0.753 0.451	16.61 ± 10.70	16.14	−2.748 0.006*
	No	38.64 ± 5.55	40.00		19.03 ± 5.79	20.00		21.58 ± 12.75	19.27	
Alcohol or Tobacco Use**	Yes	43.78 ± 7.58	42.00	−0.742 0.458	20.78 ± 7.03	21.00	−1.973 0.049*	19.66 ± 14.49	16.14	−0.478 0.633
	No	43.37 ± 5.99	44.00		18.40 ± 5.90	19.00		17.39 ± 10.68	16.66	

*Note:* *p < 0.05, **Mann−Whitney *U* test, ***Kruskal−Wallis test.

Health anxiety scores significantly varied according to income status, chronic illness status, perceived health status, medication use, and alcohol/tobacco consumption (*p* < 0.05). Students with chronic illnesses (23.10 ± 5.82) had higher anxiety scores than those without (18.25 ± 5.98). Similarly, those using medications (21.08 ± 6.54) or engaging in alcohol/tobacco use (20.78 ± 7.03) reported higher anxiety scores than non‐users. Post‐hoc analyses showed that students with lower income‐to‐expense ratios (19.77 ± 4.25) had higher anxiety than those with balanced financial conditions (17.14 ± 5.97), and those perceiving their health as poor (23.73 ± 9.49) had higher anxiety than those rating it as moderate (19.84 ± 5.41) or good 16.64 ± 5.74).

Health literacy scores showed significant differences based on academic department, year of study, perceived health status, and perceived usefulness of online health information (*p* < 0.05). Notably, students who found health information from the internet useful had lower health literacy scores (16.61 ± 10.70) than those who did not (21.58 ± 12.75). Post‐hoc results indicated that students from the Social Work Department (24.24 ± 11.71) had higher literacy levels than those in the Nutrition and Dietetics Department (14.12 ± 9.84). Additionally, first‐year students (19.22 ± 10.60) had higher health literacy compared to second‐year (18.45 ± 10.53) and fourth‐year students (12.81 ± 9.54).

## 4 Discussion

This study explored the levels of health literacy, health anxiety, and health perception among students in a faculty of health sciences, along with the relationships among these variables. The findings offer insight into the cognitive and emotional dimensions of students' health awareness and behaviour, contributing to the existing literature on university student health.

Overall, the study revealed that students' health literacy levels were generally inadequate. This is consistent with national studies in Turkey reporting insufficient or limited health literacy among university students [27, 28, 29]. Health literacy is not limited to information access—it also includes the ability to interpret, evaluate, and apply health knowledge in daily life [30].

Although students may have theoretical knowledge, they may lack the skills to manage and apply this knowledge effectively in practice.

Findings showed significant differences in health literacy across academic departments and year of study. Students in Social Work had higher health literacy than those in Nutrition and Dietetics, possibly due to the broader public health and policy context emphasised in social work curricula [31, 32]. First‐year students showed higher literacy than upperclassmen, which may be explained by their exposure to more foundational health knowledge and a more generalised interest in health during early training [33, 34]. Conversely, some studies report that health literacy decreases as year of study increases, potentially due to increased critical awareness and more realistic self‐assessment [35, 36].

The significant association between perceived usefulness of online health information and health literacy supports the growing importance of digital health literacy [37, 38]. However, exposure to online information does not always guarantee accuracy, and misinformation may increase health anxiety [39]. Therefore, the ability to critically evaluate online health information represents an essential component of modern health literacy.

Students' average health anxiety scores were found to be in the low‐to‐moderate range, consistent with previous studies [40, 41, 42]. Health anxiety, often triggered by normal bodily sensations interpreted as symptoms, may be influenced by both knowledge and emotion [43, 44]. The weak but significant positive correlation between health literacy and health anxiety may reflect both directions: greater health knowledge could increase disease‐related rumination, or anxious individuals may seek out more health information [45, 46].

Psychological factors may help explain the observed associations between cognitive and emotional dimensions of health. Previous studies indicate that emotional processes influence how individuals interpret health‐related information and respond to perceived risks. For example, research has shown that psychological characteristics such as depression, emotional awareness, and emotional regulation abilities may influence individuals' behavioural responses to health‐related stimuli [13]. Similarly, emotional processes may affect how individuals perceive bodily sensations and interpret potential symptoms, potentially contributing to increased levels of health‐related concern. Individuals with greater sensitivity to emotional or physical experiences may demonstrate increased vigilance toward perceived health threats, which may partially explain the relationship between health literacy and health anxiety.

The positive association between health perception and health anxiety observed in this study suggests that individuals who are more aware of their health status may also be more attentive to potential health problems [18, 47]. Individuals who actively monitor their health may interpret physical sensations more carefully, which may increase awareness of potential risks while simultaneously contributing to increased concern. This finding supports the notion that health awareness does not always result in reduced anxiety, and that increased knowledge about health risks may sometimes increase perceived vulnerability.

Higher anxiety scores among students with chronic conditions, those using medications, or engaging in risk behaviours may reflect a heightened sense of vulnerability and threat perception [45]. Moderate health perception levels were observed overall. The positive correlation between health perception and anxiety suggests that those who are more attuned to their health may also worry more about potential problems [24, 46].

Higher levels of health perception among students whose mothers had higher levels of education suggest that parental education may indirectly influence health‐related knowledge, attitudes, and behaviours [48, 49]. Individuals raised in environments characterised by higher educational attainment may have greater exposure to health‐promoting behaviours, which may influence the development of positive health‐related beliefs. In addition, students who reported frequently reading scientific or health‐related materials demonstrated more positive health perception scores. This finding highlights the importance of proactive information‐seeking behaviours in shaping health awareness and suggests that active engagement with reliable health information sources may support the development of more positive health‐related beliefs.

Spearman correlation analysis revealed weak but statistically significant associations among health literacy, health anxiety, and health perception, suggesting partial interdependence among cognitive, emotional, and evaluative aspects of health. Given the cross‐sectional design, causality and directionality cannot be inferred. While each variable operates somewhat independently, they also contribute to shaping health‐related behaviour [50, 51, 52]. Thus, comprehensive interventions should target not only health knowledge but also psychological resilience and perception.

Previous research has emphasised that psychological characteristics such as emotion regulation ability, coping strategies, and psychological distress may influence health‐related behaviours and attitudes. Studies conducted among university students have demonstrated that emotional eating behaviours are significantly associated with psychological determinants, suggesting that emotional processes may shape behavioural responses to health‐related information. Arslan and Alataş reported that increased emotional eating and higher body mass index significantly increased the risk of binge eating disorder among university students, indicating that emotional responses may directly influence health‐related behavioural patterns and decision‐making processes [12]. Similarly, research examining attitudes toward functional foods showed that as students' basic nutrition knowledge increased, their attitudes toward functional foods became more positive; however, behavioural tendencies were also influenced by trust, perceived usefulness, and personal preferences rather than knowledge alone [13]. These findings suggest that health literacy alone may not fully explain health‐related behaviours and that psychological and behavioural determinants should also be considered when interpreting health‐related outcomes. Individuals may possess adequate health knowledge but still respond differently to health‐related risks depending on emotional sensitivity, coping styles, and behavioural tendencies. Therefore, the relationship between health literacy and health anxiety may be better understood within a broader psychosocial framework that includes both cognitive and emotional determinants.

### 4.1 Limitations

This study has several limitations that should be considered when interpreting the findings. First, the research was conducted among students enroled in the Faculty of Health Sciences at a single university, which may limit the generalisability of the results to students from other faculties, universities, or geographical regions. Second, the cross‐sectional design does not allow conclusions regarding causal relationships among health literacy, health anxiety, and health perception. Third, data were collected using self‐report instruments, which may be subject to response bias and subjective interpretation.

In addition, evaluating complex and multidimensional constructs such as health literacy, health anxiety, and health perception using a single measurement tool for each variable may not fully capture the comprehensive nature of these concepts. Psychological and behavioural factors such as coping strategies, emotional regulation, and mental health status were not included in the study and may influence health‐related outcomes.

Despite these limitations, the study has important strengths, including an adequate sample size, the use of probability‐based sampling methods, and high statistical power, which increase the reliability of the findings.

## 5 Conclusion

This study contributes to the growing literature on student health by highlighting the interconnected roles of health literacy, health anxiety, and health perception among future healthcare professionals. The findings indicate that health sciences students demonstrated generally inadequate health literacy, moderate health perception, and low‐to‐moderate health anxiety levels. Weak but statistically significant associations among these variables suggest that cognitive competencies related to health information may interact with emotional responses and subjective health evaluations.

The results emphasise that health literacy alone may not be sufficient to explain health‐related attitudes and behaviours, as psychological factors may influence how individuals interpret health information and respond to perceived health risks. These findings underscore the importance of addressing both cognitive and emotional dimensions of health awareness in educational settings.

Educational strategies that integrate health literacy development with critical evaluation skills and psychological support mechanisms may contribute to improving students' capacity to interpret health information effectively and manage health‐related concerns. Supporting multidimensional health competencies among health sciences students may enhance not only their personal well‐being but also their future professional capacity to promote evidence‐based health practices.

Future studies employing longitudinal and mixed‐method designs may provide deeper insight into the mechanisms linking health literacy, health anxiety, and health perception, thereby supporting the development of more comprehensive health promotion interventions in university settings.

## Author Contributions

Study conception and design: Akgün Yeşiltepe, Ferhat Daşbilek, Mehmet Osman Ayhan, Data collection: Akgün Yeşiltepe, Ferhat Daşbilek, Mehmet Osman Ayhan, Data analysis and interpretation: Akgün Yeşiltepe, Ferhat Daşbilek, Mehmet Osman Ayhan, Drafting of the manuscript: Akgün Yeşiltepe, Ferhat Daşbilek, Mehmet Osman Ayhan, Critical revision of the manuscript: Akgün Yeşiltepe.

## Funding

The authors have nothing to report.

## Ethics Statement

Informed consent was obtained from all participants prior to participation using a consent form. After obtaining permission from the state institution to conduct the study, ethical approval was received from Munzur University Non‐Invasive Studies Ethics Committee (Date: 28.04.2022; Approval No: E.51893). In line with the principles of the Declaration of Helsinki, consent was received from the participants.

## Consent

The authors have nothing to report.

## Conflicts of Interest

All authors certify that there are no conflicts of interest with other people or organizations that may be perceived to adversely affect or affect their work, financial, personal, or other relationships.

## Data Availability

The data that support the findings of this study are available from the corresponding author upon reasonable request.
